# Is routine antibiotic prophylaxis warranted in dental implant surgery to prevent early implant failure? – a systematic review

**DOI:** 10.1186/s12903-024-04611-0

**Published:** 2024-07-25

**Authors:** Palwasha Momand, Aron Naimi-Akbar, Margareta Hultin, Bodil Lund, Bengt Götrick

**Affiliations:** 1 Department of Orofacial Medicine, Faculty of Odontology, Malmö University, Malmö, SE-20506 Sweden; 2 Faculty of Odontology, Health Technology Assessment-Odontology (HTA-O), Malmö University, Malmö, Sweden; 3 Department of Dental Medicine, Division of Periodontology, Karolinska Institutet, Stockholm, Sweden; 4 Department of Dental Medicine, Karolinska Institute, Stockholm, Sweden; 5 Medical Unit of Plastic Surgery and Oral and Maxillofacial Surgery, Karolinska University Hospital, Stockholm, Sweden

**Keywords:** Dental implants, Antibiotic prophylaxis, Implant failure

## Abstract

**Background:**

The question of whether antibiotic prophylaxis should be administered routinely for dental implant surgery is unresolved. Despite the lack of conclusive supportive evidence, antibiotics are often administered to reduce the risk of infection, which could lead to early implant failure. Increasing antibiotic resistance is a major concern and it is therefore important to reduce the overall use of antibiotics, including in dentistry. The aim of the present systematic review and meta-analysis was to evaluate the efficacy of preoperative antibiotics in preventing early implant failure, in overall healthy patients undergoing dental implant surgery.

**Methods:**

An electronic search was undertaken of PubMed (Medline), Web of Science and the Cochrane Library up to October 1^st^, 2023, to identify randomized clinical trials (RCTs). All RCTs comparing antibiotic prophylaxis with no antibiotics/placebo in overall healthy patients receiving dental implants were included. The primary outcome was patients with early implant failure. Risk of bias was assessed, data were extracted, a meta-analysis was done, and GRADE certainty-of-evidence ratings were determined. The risk ratio (RR), the risk difference (RD) and 95% confidence intervals (CI) were estimated.

**Results:**

After removal of duplicates, 1086 abstracts were screened, and 17 articles were reviewed in full text. Seven RCTs with moderate or low risk of bias and with a total of 1859 patients and 3014 implants were included in the meta-analysis. With reference to early implant failure at patient level, the meta-analysis failed to disclose any statistically significant difference (RR: 0.66, 95% CI: 0.30-1.47) between antibiotic prophylaxis and a placebo. The risk difference was -0.007 (95% CI: -0.035-0.020) leading to a number needed to treat (NNT) of 143.

**Conclusion:**

Antibiotic prophylaxis for dental implant surgery does not seem to have any substantial effect on early implant failure (  ). The results do not support routine antibiotic prophylaxis for dental implant surgery.

## Background

Dental implants are commonly used to replace missing teeth in patients who have lost a tooth, or teeth, primarily due to dental caries, periodontal disease, or trauma [1]. While the procedure generally has a high success rate, complications can occur. The biological complications may be early or late. Early failure can be defined as loss of the implant within the first months after insertion and is usually due to lack of osseointegration [2]. Early implant failures have been attributed to bacterial contamination during implant surgery [3]. However, other factors have also been implicated, such as surgical technique, implant characteristics (size, length and surface characteristics of the dental implant), the surgeon’s experience, a history of periodontitis and smoking habits [4–6].

To reduce the risk of infection, leading to failure of osseointegration, antibiotics can be administered in conjunction with implant surgery. Initially, the recommended routine was to administer antibiotics, both pre- and postoperatively, to all patients [7]. This routine was questioned as early as 25 years ago [8] and the issue of whether antibiotic prophylaxis is of benefit to implant placement remains unresolved. To date, no placebo-controlled, randomized clinical trial (RCT) has been able to show any statistically significant association between antibiotic prophylaxis and a reduction in the rate of early implant failure [9–19]. While one explanation might be that the RCTs were underpowered, two of the RCTs included quite large sample sizes, 506 [11] and 473 patients [12] respectively: conducting even larger RCTs would probably prove impractical.

Nevertheless, in reviews and meta-analyses it has been possible to compile studies with non-significant results and to show statistically that antibiotic prophylaxis significantly reduces early implant failures in healthy patients [20–24]. However, showing that antibiotic prophylaxis leads to a statistically significant reduction in the rate of early implant failure does not necessarily mean that routine antibiotic prophylaxis is clinically relevant. There is a need to determine the risk difference of implant failure when antibiotic prophylaxis is compared with a placebo. If this is very low, then perhaps antibiotic prophylaxis should be avoided. It is well known that all use of antibiotics contributes to the development of antibiotic resistance, which is a major global concern [25, 26]. Thus each dose of antibiotics, whether in healthcare or dentistry, should be carefully considered and prescribed only if it is truly necessary.

The consequences of early implant loss must be weighed against the risks associated with unnecessary administration of antibiotics to multiple patients. In this assessment, opinions differ. Some systemic reviews [20–23, 27–30] conclude that antibiotic prophylaxis is indicated to prevent early implant failure in healthy patients, whereas others conclude that routine use of antibiotics may not be warranted in such patients [31–35]. Inconsistent conclusions and opinions about the benefit of antibiotic prophylaxis for implant surgery have contributed to difficulties in formulating clear and generally acceptable guidelines.

Because of the limited number of RCTs in the field, previous systematic reviews and meta-analyses [20–24, 27–30, 33–35] have included studies which were not placebo-controlled, or not blinded, or which for other reasons were judged to have a high risk of bias. However, new RCTs have been published [12–14] in the last two years, one of which have not been included in any systematic review to date [14]. New, well-conducted RCTs should make it possible to conduct a meta-analysis without having to include RCTs with a high risk of bias. We therefore considered that a new systematic review was warranted, which included the most recent RCT and included the aspect of certainty of evidence, which has not been included in any published systematic review to date. The present study comprises a systematic review and meta-analysis. The aim was twofold: to evaluate the efficacy of preoperative antibiotics in prevention of early dental implant failure in healthy patients and secondly, to determine the certainty of the evidence.

## Methods

The study was conducted in accordance with the Preferred Reporting Items for Systematic reviews and Meta-Analyses (PRISMA) statement [36]. The protocol for the current study was registered at: https://www.crd.york.ac.uk/prospero (ID code: CRD42021292610).

### Focused question

The focused question was: “What is the effect of antibiotic prophylaxis compared to placebo/no antibiotics in overall healthy patients undergoing dental implant surgery regarding implant failure?”

The predefined study population, intervention, comparing therapies and outcome parameters (PICO) were:

- P (population): Patients without serious health issues undergoing dental implant surgery
- I (intervention): Administration of systemic prophylactic antibiotics in conjunction with dental implant surgery
- C (comparison): Administration of a placebo, or no antibiotic therapy in conjunction with dental implant surgery
- O (outcome): Early implant failure (implants which had to be removed before prosthetic loading, due to lack of osseointegration)

### Eligibility criteria

Studies which met the following criteria were included:

#### Inclusion criteria

- Study population of at least 20 patients
- Randomized controlled trials (RCTs)
- At least 2 months’ follow-up

#### Exclusion criteria

- Studies on mini-implants or orthodontic mini-screws
- Studies on immediate implant placement at a site with apical pathology
- Studies which included patients whose medical history indicated the need for antibiotic prophylaxis prior to dental implant surgery

### Information sources and search strategies

Three electronic databases were searched: PubMed (Medline), the Cochrane Library and Web of Science, Table 1. The authors designed and undertook the searches in collaboration with information specialists at Malmö University. The searches included articles published up to October 1^st^, 2023. The review authors PM and BG carried out duplicate hand searches of the reference lists of relevant literature. Further searches were undertaken of the online databases providing information about ongoing clinical trials (clinicaltrials.gov; www.centerwatch.com/clinical-trials). An article identified by at least one of the two review authors was included for further scrutiny.

**Table 1:** Search strategies used in the databases

**Database**	**Search terms**	**References found**
PubMed (Medline)	(("randomized controlled trial"[Publication Type] OR "controlled clinical trial"[Publication Type] OR "randomized"[Title/Abstract] OR "placebo"[Title/Abstract] OR "drug therapy"[MeSH Subheading] OR "randomly"[Title/Abstract] OR "trial"[Title/Abstract] OR "groups"[Title/Abstract]) NOT ("animals"[MeSH Terms] NOT "humans"[MeSH Terms])) AND ((("dental*"[Title/Abstract] AND "implant*"[Title/Abstract]) OR ("dental*"[Title/Abstract] AND "prosthes*"[Title/Abstract]) OR ("osseointegrat*"[Title/Abstract] AND "implant*"[Title/Abstract] AND ("oral"[Title/Abstract] OR "dental"[Title/Abstract])) OR (("overdentur*"[Title/Abstract] OR "crown*"[Title/Abstract] OR "bridge*"[Title/Abstract] OR "restoration*"[Title/Abstract]) AND ("dental"[Title/Abstract] OR "oral"[Title/Abstract]) AND "implant*"[Title/Abstract]) OR "implant supported dental prosthesis"[Title/Abstract] OR ("blade"[Title/Abstract] AND "implant*"[Title/Abstract] AND ("dental"[Title/Abstract] OR "oral"[Title/Abstract])) OR ("endosseous"[Title/Abstract] AND "implant*"[Title/Abstract] AND ("dental"[Title/Abstract] OR "oral"[Title/Abstract])) OR (("dental"[Title/Abstract] OR "oral"[Title/Abstract]) AND "implant*"[Title/Abstract]) OR "Dental Implants"[MeSH Terms] OR "Dental Implantation"[MeSH Terms] OR "dental prosthesis, implant supported"[MeSH Terms] OR "Bone-Anchored Prosthesis"[MeSH Terms]) AND ("antibiotic*"[Title/Abstract] OR ("Anti-Bacterial"[Title/Abstract] AND "agent*"[Title/Abstract]) OR "penicillin*"[Title/Abstract] OR "lincosamid*"[Title/Abstract] OR "Anti-Bacterial Agents"[MeSH Terms] OR "Penicillins"[MeSH Terms] OR "Antibiotic Prophylaxis"[MeSH Terms] OR "Chemoprevention"[MeSH Terms] OR "Lincosamides"[MeSH Terms]))	635
Cochrane Library	#1: antibiotic* OR (Anti-Bacterial AND Agent*) OR penicillin* OR lincosamid* #2: (Dental* AND implant*) OR (Dental* prosthes* OR osseointegrat* AND implant*) AND (oral OR dental) OR (overdentur* OR crown* OR bridge* OR restoration*) AND (dental OR oral) AND implant* OR "implant supported dental prosthesis" OR (blade AND implant*) AND (dental OR oral) OR (endosseous AND implant*) and (dental OR oral) OR ((dental OR oral) AND implant*) Searched: #1 AND #2	332
Web of Science	#1: antibiotic* OR (Anti-Bacterial AND Agent*) OR penicillin* OR lincosamid* (All Fields) #2: (Dental* AND implant*) OR (Dental* prosthes* OR osseointegrat* AND implant*) AND (oral OR dental) OR (overdentur* OR crown* OR bridge* OR restoration*) AND (dental OR oral) AND implant* OR "implant supported dental prosthesis" OR (blade AND implant*) AND (dental OR oral) OR (endosseous AND implant*) AND (dental OR oral) OR ((dental OR oral) AND implant*) (All Fields) **#**3: ((randomised OR randomized OR randomisation OR randomisation OR placebo* OR (random* AND (allocat* OR assign*)) OR (blind* AND (single OR double OR treble OR triple)))) (All Fields) Searched: #1 AND #2 AND #3	300

*MeSH* Medical Subject Headings, used to index articles in the National Library of Medicine

### Data collection process

Initially, duplicates were removed from the database searches and the hand searches: thereafter, two of the authors (PM and BG) independently reviewed titles and abstracts of the retrieved studies for possible inclusion, according to the inclusion/exclusion criteria. In case of any ambiguity, the study was included. Selected studies were read in full text, also independently, by at least two of the five review authors. The studies were read to verify that they met the inclusion and exclusion criteria. During this process, any lack of consensus arising among the review authors was resolved by discussion. Reasons for exclusion were recorded. A data extraction form was prepared, and the review authors PM and BG were calibrated. These two review authors extracted data independently and the remaining review authors checked that the data had been extracted correctly. Only information of relevance to the present systematic review was registered. All original clinical trials had implant failure as either a primary or a secondary outcome. Other relevant data such as age, gender, number of included patients, operation technique, number of implants, type of implants, follow-up time, and type of drugs administered were also extracted from the included original clinical trials. In cases of inadequate data, trial authors were contacted to provide additional information to complete the data collection process.

### Study risk of bias assessment

After calibration, each of the five review authors independently assessed the risk of bias of each of the included studies. This was followed by a discussion among all the review authors, to reach consensus on points of difference. The risk of bias in randomized clinical trials of intervention tool (RoB-2) [37] was used to assess the studies as having low, moderate, or high risk of bias. The overall bias assessment for each study was determined by taking into consideration the results from each domain in the risk of bias tool that was used.

### Synthesis methods

Studies assessed as having low or moderate risk of bias were included in the meta-analysis. A random effects model (Hedges) was used to calculate risk ratios (RR) and risk differences (RD). Statistical heterogeneity was assessed and presented with I^2^ and Q statistics. Additional meta-analyses were made by creating two sub-groups: one comprising only the studies using amoxicillin and the other comprising all studies except for the two which included patients who underwent immediate implant placement into extraction sockets.

Amoxicillin is the most common type of prophylactic antibiotic used in dental implant surgery and amoxicillin was used in all included studies except for one study where clindamycin was used. As clindamycin is an antibiotic with completely different properties than amoxicillin, it was decided to create a subgroup that excluded the study that used clindamycin to report only the effect of amoxicillin. The decision to create a subgroup that excluded the two studies that included immediate implant placement in extraction sockets was because these studies included a method reported to have a higher risk of failure.

Stata 16 SE was used for statistical calculations.

### Certainty assessment

The Grading of Recommendations, Assessment, Development and Evaluations (GRADE) [38] approach was used to determine the certainty of evidence related to the outcome “implant failure” in the RCTs, as high, moderate, low, or very low.

## Results

### Study selection

**Fig. 1:**
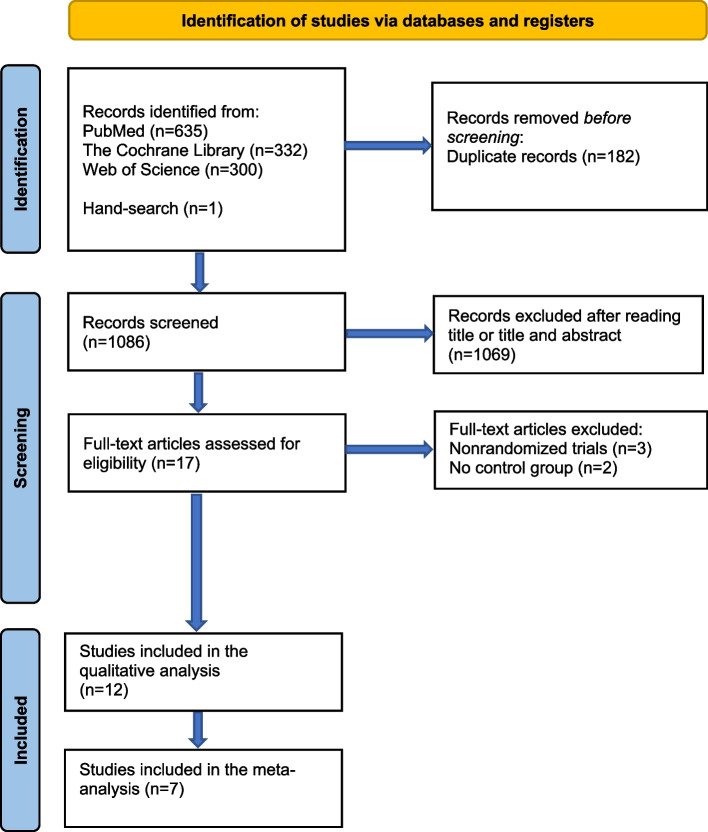
Flow chart illustrating the screening process for eligible primary studies

The search yielded a total of 1267 records. After removing 182 duplicates, 1085 titles and titles and abstracts were read and analyzed for relevancy. Of these, 1069 records were excluded, and the remaining 16 articles were read in full text. Hand-searches yielded yet another study that was read in full-text and included for further assessment. Five of these articles [39–43] were excluded because they were either not randomized, or they lacked a control group given no antibiotics or a placebo, Table 2. Hence, twelve studies were eligible for risk of bias assessment and seven were ultimately included in the meta-analysis. The flow charts presented in Fig. 1 illustrate the screening process.

**Table 2:** Comments regarding reason for exclusion of studies read in full text

**Author**	**Reason for exclusion**
Binahmed et al. (2005) [39] Canada	Not randomized
Laskin et al. (2000) [41] USA	Not randomized
Karaky et al. (2011) [40] Jordan	Not randomized
Arduino et al. (2015) [42] Italy	Lacked control group given no antibiotics/placebo
El-Kholey et al. (2014) [43] Saudi Arabia	Lacked control group given no antibiotics/placebo

### Risk of bias in studies

Table 3 presents the risk of bias in the 12 studies included in the qualitative analysis. Five were judged to have an overall high risk of bias and were therefore excluded [16–19, 44]. Comments on these studies are presented in Table 4. Four studies were considered to have a low overall risk of bias [12–15]. Three studies were considered to have a moderate overall risk of bias, due to moderate risk of bias in the domain “Conflict of Interest” [9–11]. The existence of a number of RCTs within the scope of this review enabled the exclusion of studies with a high risk of bias. Exclusion of such RCTs increases the credibility of the results of the meta-analysis.

**Table 3:**
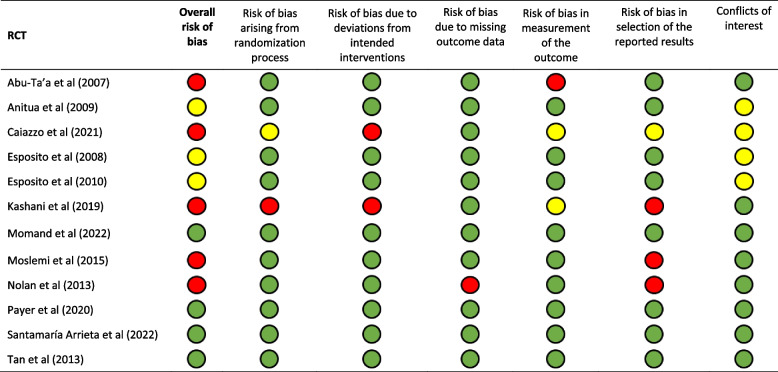
Methodological assessment of the remaining RCTs after full text assessment (*n*=12) with the Risk of Bias in RCTs of interventions (RoB-2) tool Abu-Ta’a et al. (2008) [18], Anitua et al. (2009) [9], Caiazzo et al. (2021) [49], Esposito et al. (2008) [10], Esposito et al. (2010) [11], Kashani et al. (2019) [44], Momand et al. (2022) [12], Moslemi et al. (2015) [19], Nolan et al. (2014)  [17], Payer et al. (2020) [13], Santamaría Arrieta et al. (2022) [14], Tan et al. (2016) [15]

Risk of bias:  = low

= moderate

= high

**Table 4:** Comments regarding RCTs with high risk of bias

**RCT**	**Comments**
Abu-Ta’a et al. (2008) [18] Belgium	No placebo Treatment subjects not blinded Unclear outcome measurements Unclear randomization protocol No published study protocol
Caiazzo et al. (2011) [16] Italy	No placebo Treatment subjects not blinded Unclear outcome measurements Unclear presentation of baseline data Unclear randomization process and allocation concealment
Kashani et al. (2019) [44] Sweden	No placebo Treatment subjects not blinded Unclear randomization process Unclear outcome measurements
Moslemi et al. (2015) Iran	Unclear randomization protocol Unclear definition of outcome measure No published study protocol
Nolan et al. (2013) Ireland	Methods used to produce allocation sequence not presented No presentation of number of implants placed in treatment groups Unclear definition of outcome measure Very high loss to follow-up (28 patients, 35%) No published study protocol

### Characteristics of studies included in the meta-analysis

Six of the seven studies included in the meta-analysis were multicenter RCTs [9–13, 15]. Six were conducted in Europe [9–14] and one in Asia [15]. Three studies were undertaken in private dental clinics [9–11], three mainly in university clinics [13–15], and one mainly in specialist public dental clinics [12]. Patients were recruited and treated from January 2006 to June 2021. All RCTs were double-blinded. In five RCTs, administration of 2 g of amoxicillin 1 hour prior to surgery was compared with a placebo [9–12, 15], one RCT compared 2 g of amoxicillin 1 hour prior to surgery + 500 mg thrice daily on days 1-3 after surgery, with a placebo [13] and one RCT compared 600 mg of clindamycin 1 hour prior to surgery with a placebo [14]. Three RCTs included patients who received conventional single implant placement without any bone-augmentation [9, 14, 15]. Two RCTs included a smaller number of implants inserted into fresh extraction sockets: 136 implants (19.5%) [10] and 136 implants (14.0%) [11], respectively. One RCT also included patients who underwent implant placement with simultaneous minor bone augmentation, a sinus lift, or guided bone regeneration (GBR), a total of 127 patients (29.9%) [12]. Finally, one RCT included only cases requiring implant placement with simultaneous GBR [13]. In this RCT, one study implant per patient was randomly selected. The main characteristics of the seven RCTs included in the meta-analysis are presented in Table 5.

**Table 5:** Characteristics of included RCTs with low or moderate risk of bias

**RCT**	**Population**	**Study period**	**Antibiotics**	**Placebo**	**No. of centers**	**Surgical characteristics**	**Additional information**
Anitua et al. (2009) [9]	n: 105 Age: 18-75 years Gender (m/f): 35/70 Implants inserted: 105	3 months	Amoxicillin 2 g 1 h preop	Placebo 1 h preop	8	Conventional single implant insertion: 105 (100%)	Smokers: 18 (17.1%) CHX rinse preop
Esposito et al. (2008) [10]	n: 316 Age: 18-78 years Gender (m/f): 142/174 Implants inserted: 696	4 months	Amoxicillin 2 g 1 h preop	Placebo 1 h preop	11	Immediate insertion in extraction sockets: 136 (43.0%)	Smokers: 109 (34.5%) CHX rinse preop and postop twice/day for 2 weeks
Esposito et al. (2010) [11]	n: 506 Age: 18-86 years Gender (m/f): 236/270 Implants inserted: 997	4 months	Amoxicillin 2 g 1 h preop	Placebo 1 h preop	10	Immediate insertion in extraction sockets: 136 (26.9%)	Smokers: 169 (33.4%) CHX rinse preop and postop twice/day for 2 weeks
Momand et al. (2022) [12]	n: 473 Age: 18-72 years (mean 57.4 years) Gender (m/f): 239/235 Implants inserted: 757	3-6 months	Amoxicillin 2 g 1 h preop	Placebo 1 h preop	7	Insertion with GBR: 29 (6.1%) Insertion with sinus lift: 21 (4.4%) Insertion with minor bone augmentations: 78 (16.5%)	Smokers: 86 (18.1%) CHX rinse was recommended in accordance with each operator
Payer et al. (2020) [13]	n: 236 Age: mean 46 years Gender (m/f): 125/111 Implants inserted: 236	3 months	Amoxicillin 2 g 1 h preop + 500 mg x 3 for 3 days	Placebo 1 h preop + x 3 for 3 days	7	Insertion with GBR: 236 (100%)	Smokers: 22 (9.3%) CHX rinse preop and postop 2 times/day for 2 weeks
Santamaría Arrieta et al. (2022) [14]	n: 62 Age: mean 48.6 years Gender (m/f): 22/40 Implants inserted: 62	2 months	Clindamycin 600 mg 1 h preop	Placebo 1 h preop	1	Conventional single implant insertion: 62 (100%)	Smokers: 13 (21.0%) CHX rinse: unknown
Tan et al. (2013)	n: 161 Age: mean 47.1 years Gender (m/f): 88/73 Implants inserted: 161	2 months	Amoxicillin 2 g 1 h preop	Placebo 1 h preop	7	Conventional single implant insertion: 161 (100%)	Smokers: 9.3% CHX rinse preop

The outcome variable “early implant failure” refers to the loss of an implant within the first few months after placement and before loading with the supraconstruction. [45] Most studies refer to an initial healning period of three to six months for evaluation of early implant failure, however is has been shown that the initial process of soft and hard tissue integration following implant installation typically requires 6-12 weeks. [6, 46, 47]. The timing of early implant failure was not clearly reported in any of the included RCTs. Implant stability was tested at the final follow-up which took place 3-6 months [12], 4 months [10, 11] 3 months [9, 13], 2 months [14, 15] after placement.

With respect to the outcome measurement, there was some degree of heterogeneity among the included RCTs. The following definitions were used to consider early implant failure: Implant survival measured by testing the stability [9], implant mobility measured manually and/or any infection dictating implant removal [10, 11], implants lost or low implant stability [12], implant stability (Yes/No) [13], loss or removal of an implant (peri-implant radiolucency, manual mobility, or low implant stability) [14], implant stability [15]. In all, 1859 patients and 3014 implants were analyzed. Implant failure was observed in a total of 51 patients (2.7%).

### Results of included studies

In the seven included studies, 929 patients received antibiotics and 930 were given placebos. Early implant failures occurred in 20 (2.2%) patients in the antibiotic group and 31 (3.3%) in the placebo group. The implant failure outcomes in the individual RCTs are presented in Table 6. In four of the RCTs the implant failure rate was lower in the groups given antibiotic prophylaxis [10–12, 15]. In two of the RCTs the implant failure rate was lower in the group receiving placebos [13, 14] and in one of the RCTs, the rate of implant failure was the same in both groups [9]. Overall, implant failure rates were very low and did not exceed 6.5% in any group, antibiotic or placebo. Two of the RCTs measured PROMs (Patient Reported Outcome Measures): pain and/or quality of life [13, 15]. Individually, none of the seven RCTs reported any statistical difference in the outcomes “early implant failure” or “postoperative infection”.

**Table 6:** Outcome (patients with implant failure) of included RCTs with low or moderate risk of bias

**RCT**	**Antibiotics**	**Placebo**	**Summary of conclusions according to the authors**
Anitua et al. (2009) [9] Spain	2 of 52 patients (3.8%)lost implants	2 of 53 patients (3.8%) lost implants	Antibiotic prophylaxis may not be needed. No statistically significant difference between groups (*p* > 0.05).
Esposito et al. (2008) [10] Italy	2 of 158 patients (1.3%) lost implants	8 of 158 patients (5.1%) lost implants	It might be advisable to routinely administer antibiotic prophylaxis. No statistically significant difference between groups (*p* > 0.05).
Esposito et al. (2010) [11] Italy	5 of 252 patients (2.0%) lost implants	12 of 254 patients (4.7%) lost implants	It might be advisable to routinely administer antibiotic prophylaxis. No statistically significant difference between groups (*p* > 0.05).
Momand et al. (2022) [12] Sweden	6 of 238 patients (2.5%) lost implants	7 of 235 patients (3.0%) lost implants	The effect of antibiotic prophylaxis in conjunction with dental implant surgery in preventing implant loss is small and may not be clinically relevant. No statistically significant difference between groups (*p* > 0.05).
Payer et al. (2020) [13] Austria	3 of 117 patients (2.6%) lost implants	1 of 119 patient (0.8%) lost implant	Antibiotic prophylaxis did not provide additional benefits to prevention of postsurgical complications. No statistically significant difference between groups (*p* > 0.05).
Santamaría Arrieta et al. (2022) [14] Spain	2 of 31 patients (6.5%)lost implants	0 of 31 patients (0%) lost implants	Preoperative clindamycin administration in healthy adults may not reduce implant failure. No statistically significant difference between groups (*p* > 0.05).
Tan et al. (2013) Singapore	0 of 81 patients (0%) lost implants	1 of 80 patient (1.3%) lost implant	Antibiotic prophylaxis does not improve postsurgical complications. No statistically significant difference between groups (*p* > 0.05).

### Results of synthesis

**Fig. 2:**
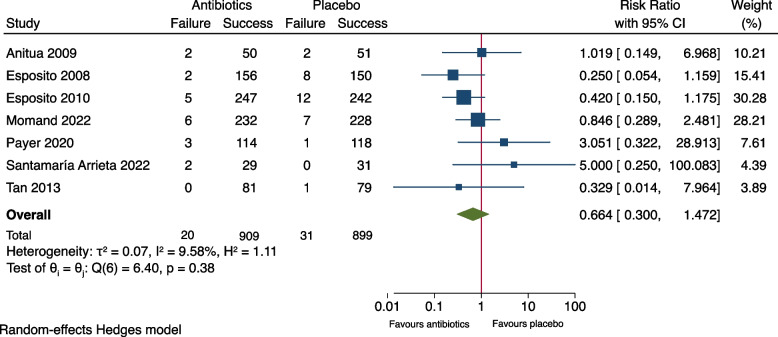
Forrest plot (risk ratio) of comparison between treatment with antibiotic prophylaxis and placebo in all RCTs using the outcome patients with implant failure

**Fig. 3:**
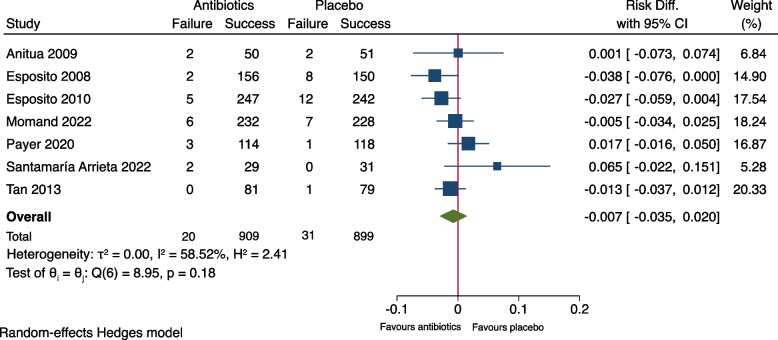
Forrest plot (risk difference) of comparison between treatment with antibiotic prophylaxis and placebo in all RCTs using the outcome patients with implant failure

**Fig. 4:**
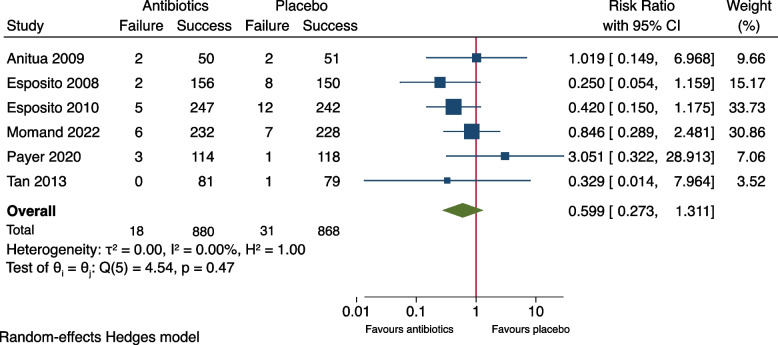
Forrest plot (risk ratio) of comparison between treatment with antibiotic prophylaxis and placebo in a subgroup (only RCTs using amoxicillin) with the outcome patients with implant failure

**Fig. 5:**
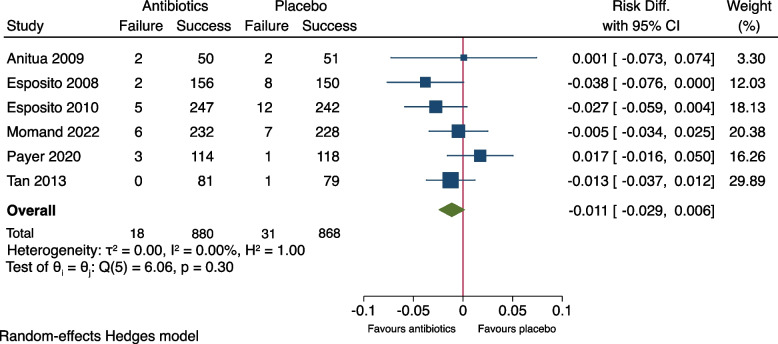
Forrest plot (risk difference) of comparison between treatment with antibiotic prophylaxis and placebo in a subgroup (only RCTs using amoxicillin) with the outcome patients with implant failure

**Fig. 6:**
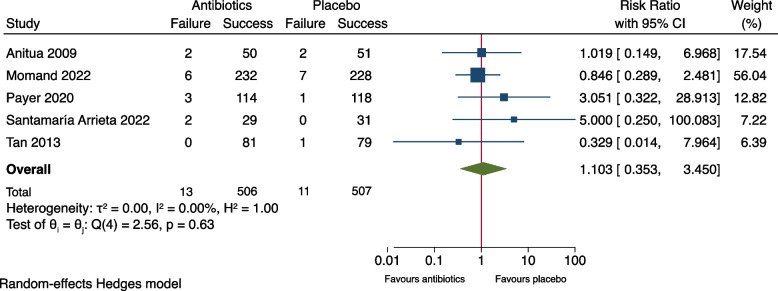
Forrest plot (risk ratio) of comparison between treatment with antibiotic prophylaxis and placebo in a subgroup (two RCTs excluded due to inclusion of patients treated with immediate insertion of implants into extraction sockets) with the outcome patients with implant failure

**Fig. 7:**
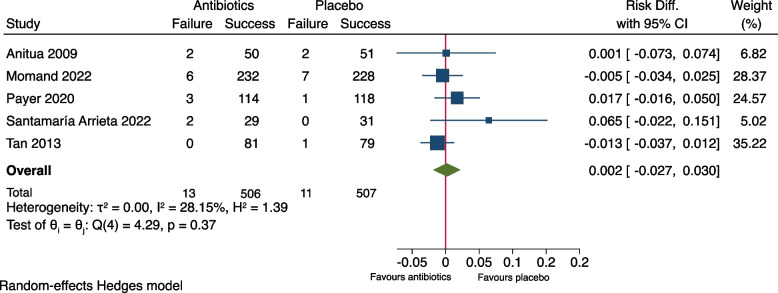
Forrest plot (risk difference) of comparison between treatment with antibiotic prophylaxis and placebo in a subgroup (two RCTs excluded due to inclusion of patients treated with immediate insertion of implants into extraction sockets) with the outcome patients with implant failure

As shown in Fig. 2, meta-analysis of the outcome measure of the seven included studies showed no significant difference between the groups (RR: 0.66, 95% CI: 0.30-1.47, *P*= 0.21). The risk difference (RD) was -0.007 (95% CI: -0.035-0.020), Fig. 3. A risk difference of -0.007 yielded an NNT of 143 (95% CI: 29-∞) to prevent implant failure in one patient. Meta-analysis of the subgroup of the six studies using amoxicillin [9–13, 15] resulted in RR: 0.60 (95% CI: 0.27-1.31) and RD: -0.011 (95% CI -0.029-0.006), Figs. 4 and 5. Meta-analysis of the subgroup comprising five studies (excluding the two [10, 11] which included patients undergoing immediate implant placement into extraction sockets) resulted in RR: 1.10 (95% CI: 0.35-3.45) and RD: 0.002 (95% CI: -0.027-0.030), Figs. 6 and 7. None of the subgroup meta-analyses showed a significant difference between the antibiotic group and the placebo group, Figs. 4, 5, 6 and 7. The results from all the meta-analyses are summarized in Table 7.

**Table 7:** Summary of the meta-analyses

**Meta-analyses**	**Number of studies**	**Number of participants**	**Number of implants**	**Risk ratio (95% CI)**	**Risk difference (95% CI)**
All studies	7	1859	3014	0.664 (0.300-1.472)	-0.007 (-0.035-0.020)
Subgroup I	6	1797	2952	0.599 (0.273-1.311)	-0.011 (-0.029-0.006)
Subgroup II	5	1037	1321	1.103 (0.353-3.450)	0.002 (-0.027-0.030)

Subgroup I: Consisting of RCTs using only amoxicillin

Subgroup II: Excluding two RCTs due to inclusion of patients treated with immediate insertion of implant into extraction sockets

### Certainty of evidence

The certainty of the scientific evidence in support of the hypothesis that “the effect of antibiotic prophylaxis in preventing implant failure is small” was moderate (  ). Table 8 presents a summary of findings.

**Table 8:** Summary of findings. Effects and certainty of evidence regarding antibiotic prophylaxis for prevention of early implant failure in health patients

**Outcome measure**	**No. of participants**	**No. of RCTs**	**Risk Ratio (95% CI)**	**Risk Difference (95% CI)**	**Certainty of evidence (GRADE)**	**Comments**
Patients with implant failure (loss of implant)	1859	7	RR: 0.66 (95% CI: 0.30-1.47)	RD: -0.007 (95% CI: -0.035-0.020)	Moderate certainty of evidence for a very small effect^1^ of antibiotic prophylaxis regarding implant failure	Transferability^2^: -1

^1^: The effect difference between the two groups was evaluated as being very small

^2^: Lack of transferability in the included RCTs as the study period varied in each study

## Discussion

This systematic review and meta-analysis included seven placebo-controlled, double-blinded RCTs assessed as having a low or moderate risk of bias. With reference to early implant failure, none of these RCTs could report any statistically significant difference between the antibiotic group and the placebo group. In two of the included RCTs, the trial authors conclude that routine administration of antibiotic prophylaxis might be advisable [10, 11]. However, in the other five studies, the trial authors conclude that antibiotic prophylaxis may not be needed [9, 12–15]. The present meta-analysis found evidence to suggest that routine use of antibiotic prophylaxis in conjunction with implant surgery to prevent early implant failures is not needed. The NNT to prevent early implant failure in one patient was 143 (95% CI: 29-∞). That the CI goes into infinity implies that antibiotic prophylaxis has no effect. In other words, routine use of antibiotic prophylaxis would likely mean that a very large number of patients would receive antibiotics unnecessarily. Most previous reviews have included RCTs with high risk of bias, likely due to the previously limited number of well-conducted RCTs in this field. Additional strengths of this review are that the meta-analysis included only placebo controlled RCTs with moderate or low risk of bias and that the certainty of evidence related to the outcome “implant failure” in the included RCTs was determined.

The results of the present review differ from those previously published in that no statistically significant difference was disclosed in early implant failure rates between patients who received antibiotic prophylaxis and patients who received a placebo. Several systematic reviews and meta-analyses reporting the effect of antibiotic prophylaxis do not discuss effectiveness and do not provide a recommendation [30, 33, 35]. However, there are exceptions: Lund et al. (2015) [31] reported an NNT of 50 to prevent a patient from losing an implant and concluded that in uncomplicated implant surgery, antibiotic prophylaxis was of no benefit [31]. Rodríguez Sánchez et al. (2018) [23], reporting an NNT of 67, concluded that antibiotic prophylaxis is effective and efficacious in preventing implant failures [23]. In 2015 the EAO consensus conference stated that antibiotic prophylaxis is not recommended for uncomplicated implant surgery [48]. A few years later, a consensus report published by the Italian Academy of Osseointegration recommended a single dose of antibiotics in uncomplicated cases [49]. These contradictory conclusions and recommendations have meant that the issue of antibiotic prophylaxis in dental implant surgery remains controversial.

The most recent review in this area [24], which included six RCTs with a total of 1504 patients, reported antibiotic prophylaxis to be statistically significant in preventing implant failure. However, three of the six included RCTs were not placebo-controlled and not blinded and another of the RCTs had a very high loss of patients to follow-up (28 patients, 35%). None of these four RCTs were included in our review, which was limited to placebo-controlled RCTs. Our review included seven RCTs with a total of 1859 patients and the meta-analysis of the outcome measure showed no significant difference between the antibiotic group and the placebo group.

In six of the RCTs included in this systematic review [9–13, 15], the antibiotic prophylaxis comprised amoxicillin 2 g, 1 hour preoperatively: this is in accordance with the routine suggested in a Cochrane systematic review by Esposito et al. (2010) [50]. In one of the included RCTs, 2 g of preoperative amoxicillin was supplemented with postoperative amoxicillin, 500 mg x 3, on days 1-3 [13]. Finally, in the last of the included RCTs, clindamycin, 600 mg was administered 1 hour preoperatively [14]. The use of amoxicillin or clindamycin 1 hour preoperatively is in accordance with European and American guidelines for the prevention of infective endocarditis associated with invasive dental procedures in high-risk individuals [51, 52]. These guidelines recommend amoxicillin 2 g 1 hour before the procedure and in patients allergic to penicillin, clindamycin 600 mg. In a recent meta-analysis of cross-sectional studies representing five different countries, it was concluded that amoxicillin was the most frequently prescribed prophylactic antibiotic for implant surgery [53]. Clindamycin is reported to be a common alternative to amoxicillin in implant surgery on patients allergic to penicillin [54, 55].

The main limitations of this review are firstly that only seven studies with a low or moderate risk of bias were identified and secondly that two RCTs with a low number of patients were included. Differences in study design are a further limitation. The timing of final follow-up, when implant stability was tested, varied among the included studies, but it is doubtful whether this would have affected the outcome. The use of chlorhexidine rinses also varied. Two of the included RCTs [9, 15] used preoperative chlorhexidine rinses, four RCTs [10–13] used chlorhexidine rinses both pre- and postoperatively, and one RCT [14] provided no information on general administration of chlorhexidine. As chlorhexidine is a bacteriostatic and bactericidal agent, this could have been a confounding factor in this review [12]. A further limitation is the inadequate reporting of the patients' periodontal condition.

Another limitation is the variation in implant placement procedures. Three RCTs included only patients treated with single implants [9, 14, 15], one RCT included only those receiving implants with simultaneous GBR [13], and one included a number of procedures (straightforward, simultaneous minor bone augmentation, simultaneous GBR, simultaneous sinus lift) [12]. However, the diversity of surgical methods is not only a limitation. It also means that the basis for the meta-analysis more closely reflects the mix of surgical methods used by dentists who undertake different types of non-complex implant surgery. Finally, another limitation is that the proportion of smokers differed between the RCTs: from 9.3% to 34%. Smoking is a risk factor for early implant failure [56] but it is unclear whether antibiotic prophylaxis can reduce this risk. In two of the included RCTs [10, 11], some patients received immediate post-extraction implants and the incidence of implant failure was greater in these patients. This observation may have led to the conclusion from the EAO Consensus Conference in 2015 that there may be a beneficial effect of antibiotic prophylaxis to cover immediate implant placement into fresh extraction sockets [48]. It is of interest to note that the two RCTs mentioned above reported a higher proportion of patients with implant failure in the placebo group (5.1% and 4.7%) than any of the other RCTs included in this meta-analysis. Moreover, in a recent systematic review by Salgado-Peralvo et al. (2021) of antibiotic therapy in conjunction with immediate implant surgery, preoperative administration of 2-3 g amoxicillin 1 hour before surgery followed by 500 mg/8 hour for five to seven days was recommended [57]. The rationale for the recommendation for extended antibiotic prophylaxis was that a tooth extracted for implant insertion should be treated as potentially infected. Under such circumstances, this is probably better described as antibiotic treatment, rather than antibiotic prophylaxis.

The overall non-significant difference between antibiotic and placebo groups with respect to the number of patients with implant failure and the high number of patients who need to be treated with antibiotic prophylaxis to prevent implant failure in one patient, mean that it seems inappropriate to recommend routine use of antibiotic prophylaxis in conjunction with implant surgery. All use of antibiotics entails a cost, a risk of side effects and a risk of increased antibiotic resistance [58]. Antibiotics should, as far as possible, be used only to treat infections and in healthy patients it should be used for prophylaxis only in exceptional cases.

## Conclusion

Based on this review and meta-analysis of results from high-quality RCTs, the benefit of antibiotic prophylaxis for implant surgery is likely to be very limited. In the context of increasing antibiotic resistance, antibiotic prophylaxis should be avoided in most cases of implant surgery. The results of this systematic review and meta-analysis could form the foundation of new and clearer clinical guidelines for antibiotic prophylaxis in implant surgery.

## Data Availability

All datasets used and/or analyzed during the current study are available from the corresponding author upon reasonable request.

## References

[1] Griggs JA. Dental Implants. Dent Clin North Am. 2017;61(4):857–71. 28886772 10.1016/j.cden.2017.06.007

[2] Charalampakis G, Leonhardt Å, Rabe P, Dahlén G. Clinical and microbiological characteristics of peri-implantitis cases: a retrospective multicentre study. Clin Oral Implants Res. 2012;23(9):1045–54. 22092339 10.1111/j.1600-0501.2011.02258.x

[3] Esposito M, Hirsch JM, Lekholm U, Thomsen P. Biological factors contributing to failures of osseointegrated oral implants. (II). Etiopathogenesis. Eur J Oral Sci. 1998;106(3):721–64. 9672097 10.1046/j.0909-8836..t01-6-.x

[4] Chrcanovic BR, Kisch J, Albrektsson T, Wennerberg A. Factors Influencing Early Dental Implant Failures. J Dent Res. 2016;95(9):995–1002. 27146701 10.1177/0022034516646098

[5] Chatzopoulos GS, Wolff LF. Dental implant failure and factors associated with treatment outcome: A retrospective study. J Stomatol Oral Maxillofac Surg. 2023;124(2):101314. 36280552 10.1016/j.jormas.2022.10.013

[6] Derks J, Håkansson J, Wennström JL, Tomasi C, Larsson M, Berglundh T. Effectiveness of implant therapy analyzed in a Swedish population: early and late implant loss. J Dent Res. 2015;94(3 Suppl):44s–51s. 25503901 10.1177/0022034514563077 PMC4541089

[7] Adell R, Lekholm U, Rockler B, Brånemark PI. A 15-year study of osseointegrated implants in the treatment of the edentulous jaw. Int J Oral Surg. 1981;10(6):387–416. 6809663 10.1016/S0300-9785(81)80077-4

[8] Gynther GW, Köndell PA, Moberg LE, Heimdahl A. Dental implant installation without antibiotic prophylaxis. Oral Surg Oral Med Oral Pathol Oral Radiol Endod. 1998;85(5):509–11. 9619664 10.1016/S1079-2104(98)90281-5

[9] Anitua E, Aguirre JJ, Gorosabel A, Barrio P, Errazquin JM, Román P, Pla R, Carrete J, de Petro J, Orive G. A multicentre placebo-controlled randomised clinical trial of antibiotic prophylaxis for placement of single dental implants. Eur J Oral Implantol. 2009;2(4):283–92. 20467604

[10] Esposito M, Cannizzaro G, Bozzoli P, Consolo U, Felice P, Ferri V, Landriani S, Leone M, Magliano A, Pellitteri G, et al. Efficacy of prophylactic antibiotics for dental implants: a multicentre placebo-controlled randomised clinical trial. Eur J Oral Implantol. 2008;1(1):23–31. 20467641

[11] Esposito M, Cannizzaro G, Bozzoli P, Checchi L, Ferri V, Landriani S, Leone M, Todisco M, Torchio C, Testori T, et al. Effectiveness of prophylactic antibiotics at placement of dental implants: a pragmatic multicentre placebo-controlled randomised clinical trial. Eur J Oral Implantol. 2010;3(2):135–43. 20623038

[12] Momand P, Becktor JP, Naimi-Akbar A, Tobin G, Götrick B. Effect of antibiotic prophylaxis in dental implant surgery: A multicenter placebo-controlled double-blinded randomized clinical trial. Clin Implant Dent Relat Res. 2022;24(1):116–24. 35075765 10.1111/cid.13068 PMC9306815

[13] Payer M, Tan WC, Han J, Ivanovski S, Mattheos N, Pjetursson BE, Zhuang L, Fokas G, Wong MCM, Acham S, Lang NP. The effect of systemic antibiotics on clinical and patient-reported outcome measures of oral implant therapy with simultaneous guided bone regeneration. Clin Oral Implants Res. 2020;31(5):442–51. 31957070 10.1111/clr.13580

[14] Santamaría Arrieta G, Rodríguez Sánchez F, Rodriguez-Andrés C, Barbier L, Arteagoitia I: The effect of preoperative clindamycin in reducing early oral implant failure: a randomised placebo-controlled clinical trial. Clin Oral Investig. 2023;27(3):1113-1122 10.1007/s00784-022-04701-9 PMC9469834 36098814

[15] Tan WC, Ong M, Han J, Mattheos N, Pjetursson BE, Tsai AY, Sanz I, Wong MC, Lang NP. Effect of systemic antibiotics on clinical and patient-reported outcomes of implant therapy - a multicenter randomized controlled clinical trial. Clin Oral Implants Res. 2014;25(2):185–93. 23347336 10.1111/clr.12098

[16] Caiazzo A, Casavecchia P, Barone A, Brugnami F. A pilot study to determine the effectiveness of different amoxicillin regimens in implant surgery. J Oral Implantol. 2011;37(6):691–6. 20553148 10.1563/AAID-JOI-D-09-00134.1

[17] Nolan R, Kemmoona M, Polyzois I, Claffey N. The influence of prophylactic antibiotic administration on post-operative morbidity in dental implant surgery. A prospective double blind randomized controlled clinical trial. Clin Oral Implants Res. 2014;25(2):252–9. 23406290 10.1111/clr.12124

[18] Abu-Ta’a M, Quirynen M, Teughels W, van Steenberghe D. Asepsis during periodontal surgery involving oral implants and the usefulness of peri-operative antibiotics: a prospective, randomized, controlled clinical trial. J Clin Periodontol. 2008;35(1):58–63. 18021264 10.1111/j.1600-051X.2007.01162.x

[19] Moslemi N, Shahnaz A, Bahador A, Torabi S, Jabbari S, Oskouei ZA. Effect of Postoperative Amoxicillin on Early Bacterial Colonization of Peri-Implant Sulcus: A Randomized Controlled Clinical Trial. J Dent Tehran Univ Med Sci. 2016;13(5):309–17. PMC5250628 28127324

[20] Canullo L, Troiano G, Sbricoli L, Guazzo R, Laino L, Caiazzo A, Pesce P. The Use of Antibiotics in Implant Therapy: A Systematic Review and Meta-Analysis with Trial Sequential Analysis on Early Implant Failure. Int J Oral Maxillofac Implants. 2020;35(3):485–94. 32406644 10.11607/jomi.7995

[21] Roca-Millan E, Estrugo-Devesa A, Merlos A, Jané-Salas E, Vinuesa T, López-López J. Systemic Antibiotic Prophylaxis to Reduce Early Implant Failure: A Systematic Review and Meta-Analysis. Antibiotics (Basel). 2021;10(6):698. 34200841 10.3390/antibiotics10060698 PMC8230529

[22] Romandini M, De Tullio I, Congedi F, Kalemaj Z, D’Ambrosio M, Laforí A, Quaranta C, Buti J, Perfetti G. Antibiotic prophylaxis at dental implant placement: Which is the best protocol? A systematic review and network meta-analysis. J Clin Periodontol. 2019;46(3):382–95. 30729548 10.1111/jcpe.13080

[23] Rodríguez Sánchez F, Rodríguez Andrés C, Arteagoitia I. Which antibiotic regimen prevents implant failure or infection after dental implant surgery? A systematic review and meta-analysis. J Craniomaxillofac Surg. 2018;46(4):722–36. 29550218 10.1016/j.jcms.2018.02.004

[24] Torof E, Morrissey H, Ball PA. Antibiotic Use in Dental Implant Procedures: A Systematic Review and Meta-Analysis. Medicina (Kaunas). 2023;59(4):713. 37109671 10.3390/medicina59040713 PMC10146405

[25] WHO. The burden of bacterial antimicrobial resistance in the WHO European region in 2019: a cross-country systematic analysis. Lancet Pub Health. 2022;7(11):e897–913. 36244350 10.1016/S2468-2667(22)00225-0 PMC9630253

[26] Antimicrobial Resistance Collaborators. Global burden of bacterial antimicrobial resistance in 2019: a systematic analysis. Lancet. 2022;399(10325):629–55. 35065702 10.1016/S0140-6736(21)02724-0 PMC8841637

[27] Esposito M, Grusovin MG, Worthington HV. Interventions for replacing missing teeth: antibiotics at dental implant placement to prevent complications. Cochrane Database Syst Rev. 2013;2013(7):Cd004152. 23904048 10.1002/14651858.CD004152.pub4 PMC6786879

[28] Ata-Ali J, Ata-Ali F, Ata-Ali F. Do antibiotics decrease implant failure and postoperative infections? A systematic review and meta-analysis. Int J Oral Maxillofac Surg. 2014;43(1):68–74. 23809986 10.1016/j.ijom.2013.05.019

[29] Kim AS, Abdelhay N, Levin L, Walters JD, Gibson MP. Antibiotic prophylaxis for implant placement: a systematic review of effects on reduction of implant failure. Br Dent J. 2020;228(12):943–51. 32591710 10.1038/s41415-020-1649-9 PMC7319948

[30] Jain A, Rai A, Singh A, Taneja S. Efficacy of preoperative antibiotics in prevention of dental implant failure: a Meta-analysis of randomized controlled trials. Oral Maxillofac Surg. 2020;24(4):469–75. 32643076 10.1007/s10006-020-00872-5

[31] Lund B, Hultin M, Tranaeus S, Naimi-Akbar A, Klinge B. Complex systematic review - Perioperative antibiotics in conjunction with dental implant placement. Clin Oral Implants Res. 2015;26(Suppl 11):1–14. 26080862 10.1111/clr.12637

[32] Park J, Tennant M, Walsh LJ, Kruger E. Is there a consensus on antibiotic usage for dental implant placement in healthy patients? Aust Dent J. 2018;63(1):25–33. 28543332 10.1111/adj.12535

[33] Braun RS, Chambrone L, Khouly I. Prophylactic antibiotic regimens in dental implant failure: A systematic review and meta-analysis. J Am Dent Assoc. 2019;150(6):e61–91. 31010572 10.1016/j.adaj.2018.10.015

[34] Singh Gill A, Morrissey H, Rahman A. A Systematic Review and Meta-Analysis Evaluating Antibiotic Prophylaxis in Dental Implants and Extraction Procedures. Medicina (Kaunas). 2018;54(6):95. 30513764 10.3390/medicina54060095 PMC6306745

[35] Chrcanovic BR, Albrektsson T, Wennerberg A. Prophylactic antibiotic regimen and dental implant failure: a meta-analysis. J Oral Rehabil. 2014;41(12):941–56. 25040894 10.1111/joor.12211

[36] Page MJ, McKenzie JE, Bossuyt PM, Boutron I, Hoffmann TC, Mulrow CD, Shamseer L, Tetzlaff JM, Akl EA, Brennan SE, et al. The PRISMA 2020 statement: an updated guideline for reporting systematic reviews. BMJ. 2021;372:n71. 33782057 10.1136/bmj.n71 PMC8005924

[37] Sterne JAC, Savović J, Page MJ, Elbers RG, Blencowe NS, Boutron I, Cates CJ, Cheng HY, Corbett MS, Eldridge SM, et al. RoB 2: a revised tool for assessing risk of bias in randomised trials. BMJ. 2019;366:l4898. 31462531 10.1136/bmj.l4898

[38] Zeng L, Brignardello-Petersen R, Hultcrantz M, Siemieniuk RAC, Santesso N, Traversy G, Izcovich A, Sadeghirad B, Alexander PE, Devji T, et al. GRADE guidelines 32: GRADE offers guidance on choosing targets of GRADE certainty of evidence ratings. J Clin Epidemiol. 2021;137:163–75. 33857619 10.1016/j.jclinepi.2021.03.026

[39] Binahmed A, Stoykewych A, Peterson L. Single preoperative dose versus long-term prophylactic antibiotic regimens in dental implant surgery. Int J Oral Maxillofac Implants. 2005;20(1):115–7. 15747682

[40] Karaky AE, Sawair FA, Al-Karadsheh OA, Eimar HA, Algarugly SA, Baqain ZH. Antibiotic prophylaxis and early dental implant failure: a quasi-random controlled clinical trial. Eur J Oral Implantol. 2011;4(1):31–8. 21594217

[41] Laskin DM, Dent CD, Morris HF, Ochi S, Olson JW. The influence of preoperative antibiotics on success of endosseous implants at 36 months. Ann Periodontol. 2000;5(1):166–74. 11885177 10.1902/annals.2000.5.1.166

[42] Arduino PG, Tirone F, Schiorlin E, Esposito M. Single preoperative dose of prophylactic amoxicillin versus a 2-day postoperative course in dental implant surgery: A two-centre randomised controlled trial. Eur J Oral Implantol. 2015;8(2):143–9. 26021225

[43] El-Kholey KE. Efficacy of two antibiotic regimens in the reduction of early dental implant failure: a pilot study. Int J Oral Maxillofac Surg. 2014;43(4):487–90. 24183737 10.1016/j.ijom.2013.09.013

[44] Kashani H, Hilon J, Rasoul MH, Friberg B. Influence of a single preoperative dose of antibiotics on the early implant failure rate. A randomized clinical trial. Clin Implant Dent Relat Res. 2019;21(2):278–83. 30838799 10.1111/cid.12724

[45] Esposito M, Hirsch JM, Lekholm U, Thomsen P. Biological factors contributing to failures of osseointegrated oral implants. (I). Success criteria and epidemiology. Eur J Oral Sci. 1998;106(1):527–51. 9527353 10.1046/j.0909-8836..t01-2-.x

[46] Abrahamsson I, Berglundh T, Linder E, Lang NP, Lindhe J. Early bone formation adjacent to rough and turned endosseous implant surfaces. An experimental study in the dog. Clin Oral Implants Res. 2004;15(4):381–92. 15248872 10.1111/j.1600-0501.2004.01082.x

[47] Alsaadi G, Quirynen M, Komárek A, van Steenberghe D. Impact of local and systemic factors on the incidence of oral implant failures, up to abutment connection. J Clin Periodontol. 2007;34(7):610–7. 17433044 10.1111/j.1600-051X.2007.01077.x

[48] Klinge B, Flemming T, Cosyn J, De Bruyn H, Eisner BM, Hultin M, Isidor F, Lang NP, Lund B, Meyle J, et al. The patient undergoing implant therapy. Summary and consensus statements. The 4th EAO Consensus Conference 2015. Clin Oral Implants Res. 2015;26(Suppl 11):64–7. 26385621 10.1111/clr.12675

[49] Caiazzo A, Canullo L, Pesce P. Consensus Report by the Italian Academy of Osseointegration on the Use of Antibiotics and Antiseptic Agents in Implant Surgery. Int J Oral Maxillofac Implants. 2021;36(1):103–5. 33600529 10.11607/jomi.8264

[50] Esposito M, Grusovin MG, Loli V, Coulthard P, Worthington HV. Does antibiotic prophylaxis at implant placement decrease early implant failures? A Cochrane systematic review. Eur J Oral Implantol. 2010;3(2):101–10. 20623035

[51] Wilson W, Taubert KA, Gewitz M, Lockhart PB, Baddour LM, Levison M, Bolger A, Cabell CH, Takahashi M, Baltimore RS, et al. Prevention of infective endocarditis: guidelines from the American Heart Association: a guideline from the American Heart Association Rheumatic Fever, Endocarditis, and Kawasaki Disease Committee, Council on Cardiovascular Disease in the Young, and the Council on Clinical Cardiology, Council on Cardiovascular Surgery and Anesthesia, and the Quality of Care and Outcomes Research Interdisciplinary Working Group. Circulation. 2007;116(15):1736–54. 17446442 10.1161/CIRCULATIONAHA.106.183095

[52] Habib G, Lancellotti P, Antunes MJ, Bongiorni MG, Casalta JP, Del Zotti F, Dulgheru R, El Khoury G, Erba PA, Lung B, et al. 2015 ESC Guidelines for the management of infective endocarditis: The Task Force for the Management of Infective Endocarditis of the European Society of Cardiology (ESC). Endorsed by: European Association for Cardio-Thoracic Surgery (EACTS), the European Association of Nuclear Medicine (EANM). Eur Heart J. 2015;36(44):3075–128. 26320109 10.1093/eurheartj/ehv319

[53] Rodríguez Sánchez F, Arteagoitia I, Teughels W, Rodríguez Andrés C, Quirynen M. Antibiotic dosage prescribed in oral implant surgery: A meta-analysis of cross-sectional surveys. PLoS ONE. 2020;15(8):e0236981. 32810135 10.1371/journal.pone.0236981 PMC7446810

[54] Salgado-Peralvo AO, Kewalramani N, Peña-Cardelles JF, Mateos-Moreno MV, Monsalve-Guil L, Jiménez-Guerra Á, Ortiz-García I, Velasco-Ortega E. Preventive Antibiotic Prescribing Habits among Professionals Dedicated to Oral Implantology: An Observational Study. Antibiotics (Basel). 2021;10(3):301. 33799411 10.3390/antibiotics10030301 PMC7999193

[55] Williams RGM. Antibiotic prophylaxis during dental implant placement in the UK. Br Dent J. 2020;229(12):787–92. 33339929 10.1038/s41415-020-2352-6

[56] Chrcanovic BR, Albrektsson T, Wennerberg A. Smoking and dental implants: A systematic review and meta-analysis. J Dent. 2015;43(5):487–98. 25778741 10.1016/j.jdent.2015.03.003

[57] Salgado-Peralvo AO, Peña-Cardelles JF, Kewalramani N, Mateos-Moreno MV, Jiménez-Guerra Á, Velasco-Ortega E, Uribarri A, Moreno-Muñoz J, Ortiz-García I, Núñez-Márquez E, Monsalve-Guil L. Preventive Antibiotic Therapy in the Placement of Immediate Implants: A Systematic Review. Antibiotics (Basel). 2021;11(1):5. 35052882 10.3390/antibiotics11010005 PMC8773177

[58] Bell BG, Schellevis F, Stobberingh E, Goossens H, Pringle M. A systematic review and meta-analysis of the effects of antibiotic consumption on antibiotic resistance. BMC Infect Dis. 2014;14:13. 24405683 10.1186/1471-2334-14-13 PMC3897982

